# Moral Judgments on Short-Term Sexual Behaviors among Chinese College Students: Exploring the Roles of Gender and Physical Attractiveness

**DOI:** 10.3389/fpsyg.2017.00191

**Published:** 2017-02-13

**Authors:** Qianguo Xiao, Aijuan Li, Yi Zhu

**Affiliations:** ^1^Laboratory of Emotion and Mental Health, Chongqing University of Arts and SciencesChongqing, China; ^2^School of Education, Inner Mongolia Normal UniversityHohhot, China; ^3^College of Humanities, Inner Mongolia University of TechnologyHohhot, China; ^4^College of Psychology and Sociology, Shenzhen UniversityShenzhen, China

**Keywords:** physical attractiveness, moral judgment, short-term sexual behaviors, gender, Chinese culture

## Abstract

This study primarily investigated the effects of gender and physical attractiveness on moral judgments on three typical kinds of short-term sexual behaviors (short-term fling, one-night stand, and hookup) in the Chinese culture context. A total of 120 university student subjects were presented with a series of stereotypically physically attractive (versus physically unattractive) photos before they rated the extent to which each of the three short-term sexual behaviors are morally acceptable. The results showed that male students judged all three behaviors to be more morally acceptable than female students did. Further analyses showed that this gender difference was moderated by the level of physical attractiveness. Under the high attractiveness condition, short-term flings and hookups were judged more morally acceptable by male students than by female students, but this gender difference was not significant under the low attractiveness condition. However, with regard to one-night stands, the data showed that male students judged this type of behavior to be more morally acceptable than did female students under the low attractiveness condition, while this gender difference was not significant under the high attractiveness condition. Thus, these findings further our understanding of how Chinese young people view different types of short-term sexual behaviors, and provide novel evidence regarding how physical attractiveness influences people’s moral judgments on short-term sexual behaviors.

## Introduction

Moral virtues are sexually attractive and considered to be powerful tools in assisting people to choose a “high quality" spouse (Miller, 2007). Many of the virtues of human beings were considered to have evolved into costly signals through sexual selection (Darwin, 1888; Roberts, 1998; Miller, 2007): in essence, the unique moral virtues in human sexual selection could be used as reliable fitness indicators (Miller, 2007). However, the sexual attraction of morality does not seem to be as strong and stable as physical attractiveness. A body of research suggests that humans have an innate tendency to choose a spouse with high physical attractiveness (Buss, 1985; Baker and Bellis, 1994; Hatfield and Sprecher, 1995; Oda, 2001; Jonason et al., 2011), and that both men and women tend to value physical attractiveness, especially in a short-term relationship (Fletcher et al., 2004; Li and Kenrick, 2006; Li et al., 2011), whereas virtues (e.g., marital commitment) are valued only when people are seeking a stable and serious long-term relationship (Urbaniak and Kilmann, 2003). Thus, the existing literature raises the interesting question of whether bodily attractiveness could influence people’s moral judgments on short-term sexual relationships (e.g., short-term flings).

An examination of the existing literature shows that answers to this question are scant, and that a large body of research has been devoted to exploring the gender difference in preferences for short-term sexual relationships. Though both men and women tend to prioritize physical attractiveness in short-term mates (Regan et al., 2000; Greitemeyer, 2005; Li and Kenrick, 2006; Li et al., 2011, 2013), men place more emphasis on physical attractiveness than women (Wiederman and Dubois, 1998; Li and Kenrick, 2006; Chang et al., 2011), have higher intent to engage in short-term sexual behaviors (Buss and Schmitt, 1993; Schmitt et al., 2001), and have lower standards for mate choice in short-term sexual relationships (Kenrick et al., 1990; Li and Kenrick, 2006). This evidence indicates that men are more oriented toward short-term mating and place more weight on physical attractiveness than women. As such, it is possible that this gender difference in preference for physical attractiveness in short-term mates may also manifest itself in a gender difference in moral judgments on short-term sexual behaviors.

The present research investigates how bodily attractiveness affects moral judgments on short-term sexual behaviors in the Chinese context. Cultural value and social beliefs influence individuals’ attitudes toward sex, which further affect their sexual behaviors (Bhavsar and Bhugra, 2013). Many cultures have traditionally paid special attention to sexual morality, viewing sex work or prostitution as immoral or sinful [e.g., in Christian culture, Buddhist culture (Peach, 2005), and in Confucian culture]. In the Chinese context, despite a profound social revolution over the past decades, a relatively conservative sexual culture still persists in China today (Tang et al., 1997; Higgins et al., 2002). Chinese sexuality is based mainly on the Confucian and Taoist traditions, which emphasize procreation and social order and discourage sex for pleasure and extramarital affairs for both genders (Ng and Lau, 1990; Juan and Matsumura, 1991; Wen, 1995). This sex culture not only has a profound impact on social policies (e.g., sex work is morally unacceptable and illegal in Mainland China), but also influences people’s mate-selection preferences (Higgins et al., 2002), sexual behaviors, and attitudes toward extramarital affairs, homosexuality, and short-term sexual behaviors (Ouyang, 2010).

During recent decades, Mainland China has witnessed a dramatic change in various aspects of its people’s social lives, including the change in values related to sexual relationships, such as a decrease in the importance of virginity and an increase in good expected income (Chang et al., 2011). Despite these changes, the evolved mate preferences seem to be invariant. For instance, Chang et al. (2011) compared modern Chinese with Chinese studied 25 years earlier and found that gender differences in mate preferences for cues to fertility (e.g., youth, physical attractiveness) and resources (e.g., status) remained unchanged. Therefore, based on the literature discussed above, we could expect men to judge short-term sexual behaviors to be more morally acceptable than women, and that physical attractiveness could moderate this gender difference. To test this hypothesis, we presented Chinese participants with physically attractive photos (versus less attractive photos) and then asked them to rate three short-term sexual behaviors, namely, short-term fling (referred to the case that a married person engages in a short-term sexual activity with someone other than his or her spouse), one-night stand (referred to the case that a person pays for sexual activity or engages in sexual relations in exchange for money), and hookup (referred to the case that a person engages in sexual activity with a friend known through communication network).

## Materials and Methods

### Participants

In total, 120 Chinese undergraduate students (60 males and 60 females, *M*_age_ = 20.03 years, *SD* = 1.17) volunteered to participate in this study, for which they were paid five RMB (approximately equal to 0.8 USD). This study used a 2 (gender: male vs. female) × 2 (attractiveness: high vs. low) between-subjects design. The participants were randomly assigned to one of the four groups with 15 males and 15 females in each group. The study was approved by the Psychology Ethics Committee, and all the participants provided written informed consent prior to participating in the study.

### Experimental Materials

**FIGURE 1:**
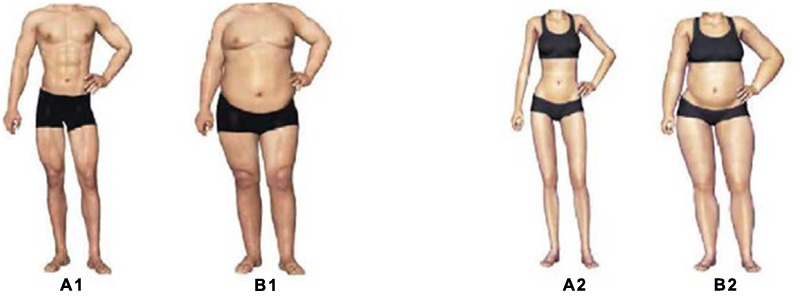
**Sample of high and low attractiveness bodies used in this study. A1** and **A2** are high attractiveness bodies, and **B1** and **B2** are low attractiveness bodies.

A prior study showed that presenting participants with attractive opposite sex induced a romantic mindset which affected their decisions (Griskevicius et al., 2007). Following this logic, we presented the participants with photos of physically attractive opposite sex bodies. We created 100 photos (238 × 350 pixels in size; *N*_male_ = 50, *N*_female_ = 50) using Adobe Photoshop based on a male and a female photo selected from the internet. The male and female bodies appeared in these photos varied in terms of waist-hip ratio (Singh, 1993; Henss, 2000) and body mass index (Tovée et al., 1998; Rosenblum and Lewis, 1999). To control the impact of race, the photos only included bodies (i.e., without a head) which had the same skin color (yellow skin which is typical in Eastern Asia). Following the process of a previous experiment (Schweitzer, 1999), another 60 undergraduate students (*N*_male_ = 30, *N*_female_ = 30) from the same university were invited to rate the attractiveness of the bodies in these photos on a 10-point scale (1 = *Extremely unattractive*, 10 = *Extremely attractive*). Based on these students’ attractiveness ratings, 20 male and 20 female highest-scoring photos and 20 male and 20 female lowest-scoring photos were selected as experimental stimuli (**Figure 1**). Results from a pair-samples *t*-test showed that the difference in the attractiveness rating between the high attractiveness and the low attractiveness group was significant (*M*_male_ ±*SD* = 7.96 ± 1.92 in the high attractiveness group, *M*_male_ ±*SD* = 2.12 ± 1.62 in the low attractiveness, *t*(58) = 12.73, *p* < 0.01; *M*_female_ ±*SD* = 7.40 ± 1.48 in the high attractiveness group, *M*_female_ ±*SD* = 3.92 ± 1.86 in the low attractiveness group, *t*(58) = 18.4, *p* < 0.01). This result suggested that these photos were appropriate for current study.

### Experimental Process

The experiment was conducted in a computer lab. The subjects participated in the experiment in groups of 3–6. Upon arrival, each subject was randomly assigned a subject number and escorted to an individual cubicle where they were seated in front of a computer screen and were not allowed to communicate with the other participants. At the outset of the experiment, they were informed that the study was about their attitudes toward social issues. They were instructed to focus on their computer screen, on which a series of photos were displayed.

Because the purpose of the current study was to examine how physical attractiveness might influence people’s moral judgments on short-term sexual behaviors, 20 photos of the opposite sex were shown to both male and female participants consecutively with 1-s display duration, and each of the 20 photos were displayed three times. After viewing all the photos, participants were instructed to complete a questionnaire on which they rated three items with each representing one type of short-term sexual behavior. More specifically, participants first read the definition of a short-term fling (“A *short-term fling* refers to the case that a married person engages in a short-term sexual activity with someone other than his or her spouse.”), a one-night stand (“A *one-night stand* refers to the case that a person pays for sexual activity or engages in sexual relations in exchange for money”), and a hookup (“A *hookup* means to engage in sexual activities with a friend known through communication network.”) and then rated the extent to which they considered the behaviors to be morally right on a 5-point Likert-style scale ranging from 1 (*Absolutely morally wrong*) to 5 (*Absolutely morally right*).

## Results

To analyze our data, we performed analysis of variance (ANOVA) with gender and physical attractiveness as independent variables and moral judgments on the three short-term sexual behaviors as dependent variables, respectively. Descriptive statistics for moral judgments on the three short-term sexual behaviors under high and low bodily attractiveness were presented in **Table 1** (see **Supplementary Materials** for data).

**Table 1:** The descriptive statistics for moral judgments on three short-term sexual behaviors under high and low bodily attractiveness.

	Gender	Attractiveness	*M* ±*SD*	*t*	*F*_gender_
Short-term fling	male	low	1.83 ± 0.87	0.14	9.24^∗∗^
		high	1.80 ± 0.96		
	female	low	1.47 ± 0.68	1.13	
		high	1.30 ± 0.53		
One-night stand	male	low	2.30 ± 0.83	-1.37	14.98^∗∗^
		high	2.60 ± 0.85		
	female	low	1.40 ± 0.49	-6.55^∗∗^	
		high	2.43 ± 0.77		
Hookup	male	low	2.33 ± 0.95	-2.19^∗^	19.35^∗∗^
		high	2.80 ± 0.66		
	female	low	1.93 ± 0.94	0.55	
		high	1.80 ± 0.88		

*^∗^ < 0.05; ^∗∗^ < 0.01.*

**FIGURE 2:**
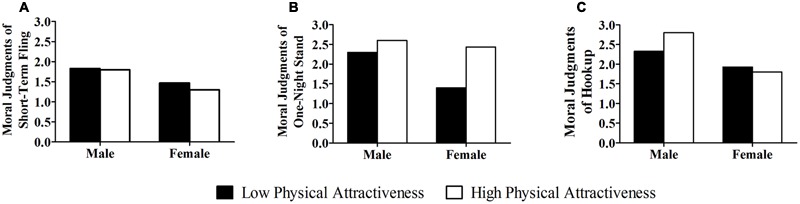
**Mean moral judgments on (A)** short-term fling, **(B)** one-night stand, and **(C)** hookup as a function of gender and physical attractiveness.

We first analyzed the effects of gender and physical attractiveness on moral judgments on a short-term fling (**Figure 2A**). The results showed a significant effect of gender, *F*(1,116) = 9.24, *p* < 0.01, $\eta_{p}^{2}$ = 0.07, suggesting that male students judged a short-term fling to be more morally acceptable than did female students. However, we observed neither a main effect of physical attractiveness, *F*(1,116) = 0.49, *p* > 0.05, nor an interaction effect between gender and physical attractiveness, *F*(1,116) = 0.22, *p* > 0.05. Further analyses showed that under the low physical attractiveness condition male students judged a short-term fling more morally acceptable than female students did, though the difference did not reach a significant level, *F*(1,58) = 3.28, *p* > 0.05, while under the high physical attractiveness condition, this gender difference was significant, *F*(1,58) = 6.20, *p* < 0.02, $\eta_{p}^{2}$ = 0.10.

We then examined the effects of gender and physical attractiveness on moral judgments on a one-night stand (**Figure 2B**). The results showed significant effects of gender, *F*(1,116) = 14.98, *p* < 0.001, $\eta_{p}^{2}$ = 0.11, and physical attractiveness, *F*(1,116) = 23.41, *p* < 0.001, $\eta_{p}^{2}$ = 0.17. Interestingly, the results also showed a significant interaction effect between gender and physical attractiveness, *F*(1,116) = 7.08, *p* < 0.01, $\eta_{p}^{2}$ = 0.06. Further analyses showed that under the low physical attractiveness condition, male students judged a one-night stand more morally acceptable than did female students, *F*(1,58) = 25.63, *p* < 0.001, $\eta_{p}^{2}$ = 0.31, while under the high physical attractiveness condition this gender difference was not significant, *F*(1,58) = 0.63, *p* > 0.05, $\eta_{p}^{2}$ = 0.01.

Finally, we investigated the effects of gender and physical attractiveness on moral judgments on a hookup (**Figure 2C**). The results showed a significant main effect of gender, *F*(1,116) = 19.35, *p* < 0.001, $\eta_{p}^{2}$ = 0.14. However, the main effect of physical attractiveness was not significant, *F*(1,116) = 1.10, *p* > 0.05. The results also showed a marginally significant interaction effect between gender and physical attractiveness, *F*(1,116) = 3.56, *p* = 0.062, $\eta_{p}^{2}$ = 0.03. Further analyses showed that under the low physical attractiveness condition, the gender difference was not significant, *F*(1,58) = 2.65, *p* > 0.05, while under the high physical attractiveness condition, the gender difference was highly significant, *F*(1,58) = 24.44, *p* < 0.001, $\eta_{p}^{2}$ = 0.30.

## Discussion

The present study examined the impact of physical attractiveness on moral judgments on short-term sexual behaviors. The results showed that male students judged short-term sexual behaviors to be more morally acceptable than female students did. Further analyses showed that this gender difference was moderated by the level of physical attractiveness. Under the high attractiveness condition, a short-term fling and a hookup are judged more morally acceptable by male students than by female students, but this gender difference was not significant under the low attractiveness condition. However, with regard to a one-night stand, data showed that male students judged this behavior to be more morally acceptable than did female students under the low attractiveness condition, while this gender difference was not significant under the high attractiveness condition.

Our findings provide further insight to the understanding of how Chinese young people view short-term sexual relationships, by showing a gender difference in the moral judgments on short-term mates. According to traditional Chinese culture, loyalty and commitment to family are highly valued, while short-term sexual behaviors—such as short-term flings, one-night stands, and hookups—are viewed as immoral. For instance, previous surveys have shown that the vast majority of respondents in China show low intolerance of extramarital sex, and they place emphasis on loyalty, responsibility, and commitment to one’s spouse (Lee, 1991; Ouyang, 2010). Our finding that male students had higher moral tolerance of short-term flings, one-night stands, and hookups than female students seems consistent with prior research revealing that Chinese female college students place more emphasis on inner qualities—such as kindness and understanding (Chang et al., 2011)—and morality—such as responsibility and loyalty (Liu et al., 2004)—in their mating. Our findings also imply that male students are more prone to short-term sexual relationships, which is in line with prior literature (Buss and Schmitt, 1993; Schmitt et al., 2001; Schmitt, 2003) and findings that men have lower standards for mate choice in short-term sexual relationships (Kenrick et al., 1990; Li and Kenrick, 2006).

Moreover, this research also adds to the literature on the association between physical attractiveness and short-term sexual behaviors. One of our key findings, that male participants only judged one-night stands as more morally acceptable than did female participants in the low physical attractiveness condition, is consistent with literature suggesting that males tend to adopt a “low-standard strategy” to have more short-term sexual relationships (Buss and Schmitt, 1993; Surbey and Conohan, 2000; Schmitt et al., 2001; Schützwohl et al., 2009). This gender difference was not significant in the high attractiveness condition, suggesting that males and females show similar preferences for a bodily attractive mate when considering a one-night stand. In addition, we also observed a significant gender difference in terms of moral judgments on short-term flings and hookups only in the high attractiveness condition. These findings point to the difference between the types of short-term sexual behaviors. As we noted earlier, in the Chinese context, short-term flings and hookups are based on some level of mutual understanding and emotional exchange, while people engage in one-night stands without mutual understanding. Chinese participants’ divergent responses to the three short-term sexual behaviors might reflect the fact that their preferences for physical attractiveness are contingent on different types of short-term sexual relationships.

Finally, our research suffers from several limitations. First, our participants are not representative of the diverse student community in China. Second, our study failed to take into consideration the potential impact of participants’ sexual orientation. Though China has a relatively conservative sexual culture (Tang et al., 1997; Chang et al., 2011), rapid social transformation during recent decades has led to cultural changes in sexuality, including the growing tolerance of homosexual relations. A recent study found that 8.5% of 1,762 college students in southwestern China (i.e., Chongqing and Chengdu) reported having sexual relationships with same-sex partner(s) (Zhang et al., 2012). Because our participants were drawn from the subject pool of a university in Chongqing, the possible small proportion of homosexuals among the participants might have affected our results. Future research is thus encouraged to control for participants’ sexual orientation and to extend our findings to a homosexual group. Third, our study cannot answer how culturally defined physical attractiveness influence people’s short-term and long-term mate preferences. Sociocultural factors play important roles in defining standards of attractiveness. For example, a study showed that Japanese are more reliant on body shape as a cue for female attractiveness than Britons, suggesting the importance of the learning of mate preferences in social and cultural contexts (Swami et al., 2007). Thus it is worthwhile to conduct cross-cultural research to further reveal how culturally defined physical attractiveness might influence people’s views on short-term sexual activities. Finally, the study used a single-item measure to assess participants’ moral judgments on each short-term sexual behavior. Because the internal consistency of single-item measures cannot be estimated, the use of more reliable multiple-item scales is encouraged in future research.

## Conclusion

This study investigated the effects of gender and physical attractiveness on participants’ moral judgments on short-term sexual behaviors in the Chinese context. Under the influence of traditional Chinese culture, people are expected to place more emphasis on morality (e.g., loyalty and responsibility) than on physical attractiveness in their mating decisions. However, our experiment found a gender difference in moral judgments on three types of short-term sexual behaviors (short-term fling, one-night stand, and hookup), and this gender effect was found to be moderated by physical attractiveness. Our findings provide novel evidence regarding how Chinese young people view short-term sexual behaviors.

## Ethics Statement

This study was carried out in accordance with the recommendations of Academic Committee of Chongqing University of Arts and Sciences with written informed consent from all subjects. All subjects gave written informed consent in accordance with the Declaration of Helsinki. The protocol was approved by the Academic Committee of Chongqing University of Arts and Sciences.

## Author Contributions

QX and AL conceived of the study and collected the data, QX and YZ analyzed the data, and QX and YZ wrote the paper.

## Conflict of Interest Statement

The authors declare that the research was conducted in the absence of any commercial or financial relationships that could be construed as a potential conflict of interest.

## Supplementary Material

DATA SHEET 1 **Raw Data**.
